# A novel oral immunotherapy strategy using transgenic barley induces *Culicoides* allergen-specific immune responses in horses

**DOI:** 10.3389/fimmu.2026.1774358

**Published:** 2026-02-10

**Authors:** Fenja Johanna von der Höden, Sara Björk Stefansdottir, Sigurbjörg Torsteinsdottir, Vilhjalmur Svansson, Jon Mar Björnsson, Bettina Wagner, Dorontina Mahmuti, Eliane Marti, Sigridur Jonsdottir

**Affiliations:** 1 Institute for Experimental Pathology, Biomedical Center, University of Iceland, Keldur, Reykjavik, Iceland; 2 Faculty of Medicine, University of Iceland, Reykjavik, Iceland; 3 ORF Genetics, Kópavogur, Iceland; 4 Department of Population Medicine and Diagnostic Sciences, College of Veterinary Medicine, Cornell University, Ithaca, NY, United States; 5 Clinical Immunology Group, Division of Neurological Science, Department of Clinical Research-Veterinary Public Health (VPH), Vetsuisse Faculty, University of Bern, Bern, Switzerland

**Keywords:** allergy, *Culicoides midges*, horse (*Equus caballus*), insect bite hypersensitivity, oral immunotherapy (OIT), transgenic barley

## Abstract

**Introduction:**

Insect bite hypersensitivity (IBH) is a seasonal, IgE-mediated allergic dermatitis of horses caused by salivary gland proteins of biting midges (*Culicoides* spp.). Current management relies on relief of clinical signs and on physical protection. In a previous pilot study, healthy horses were fed transgenic barley expressing a *Culicoides* allergen via a special spiral bit, which successfully induced allergen-specific antibody responses. Building on this concept, the present study aimed to evaluate a more practical feeding approach, delivering transgenic barley expressing the major *Culicoides* allergen Cul o 2p in a feed-compatible paste administered from buckets.

**Methods:**

Twelve naïve Icelandic horses were randomized into treatment (n=6) and control (n=6) groups. The treatment group received 11.44 g of the major *Culicoides* allergen Cul o 2p/horse across three feeding phases. Serum and saliva were analyzed for Cul o 2p-specific antibodies (IgG1, IgG4/7, IgG5, IgA, IgE) by ELISA. IgE-blocking capacity was assessed in pooled serum, and cytokine responses (IL-10, IL-4, IFN-γ) measured after *in vitro* re-stimulation of peripheral blood mononuclear cells (PBMCs).

**Results:**

Four of six treated horses produced Cul o 2p-specific IgG1, IgG4/7, and IgA in both serum and saliva. The induced antibodies could partly inhibit IgE binding. Elevated secretion of IFN-γ and IL-10 but no IL-4 secretion was observed in supernatants of re-stimulated PBMCs in treated horses compared to controls, reaching statistical significance for IFN-γ. No Cul o 2p-specific IgE was detected, and no adverse clinical reactions occurred during treatment.

**Conclusion:**

The feed-based approach using transgenic barley paste induced Cul o 2p-specific humoral and cellular immune responses in naïve horses, supporting its potential as a scalable and field-feasible platform for prophylactic and therapeutic applications in IBH. Clinical trials in IBH-affected horses are warranted to assess efficacy.

## Introduction

Insect bite hypersensitivity (IBH) is the most common allergic skin disease of horses, manifesting as a seasonal, intensely pruritic dermatitis primarily caused by bites of *Culicoides* spp. midges and mediated by an immunoglobulin E (IgE) type I hypersensitivity reaction (1–3). Clinical signs include severe pruritus, papular and crusted skin lesions, alopecia, and secondary infections, with the distribution of lesions reflecting the preferred feeding sites of the insects, such as the mane, tail, and dorsal or ventral midline (1, 4).

Icelandic horses provide a unique model for IBH research because *Culicoides* species feeding on horses are not endemic to Iceland, rendering these horses naïve to the relevant salivary allergens prior to export (5, 6). Upon introduction to *Culicoides*-infested environments, over 50% of Icelandic born horses can develop IBH, in stark contrast to a prevalence of only about 3–10% in European-born Icelandic horses (5, 7). This high disease prevalence is similarly observed in the United States, where a longitudinal cohort study reported IBH development in 62.5% of adult Icelandic horses following import and first allergen exposure (8).

During feeding, *Culicoides* inject a complex mixture of salivary gland proteins into the horse skin, which triggers Th2-mediated immune responses, drives IgE class-switch recombination and the differentiation of IgE-secreting plasmablasts and plasma cells, leading to the release of inflammatory mediators including histamine and sulfidoleukotrienes (3, 9, 10). Recent molecular profiling has identified nine major allergens from *Culicoides obsoletus*, showing D7-related/odorant-binding proteins (OBP) such as Cul o 2p to be highly immunogenic in affected horses (11).

Plant-based expression systems, such as barley, offer some advantages for production of recombinant allergens including eukaryotic post-translational modifications, high protein yields, long-term stability, negligible endotoxin content, as well as the absence of organic matter from animal origin (12, 13). Barley-expressed recombinant proteins have demonstrated comparable or superior immunoassay performance relative to those produced in *E. coli* or insect cells (14, 15).

Allergen-specific immunotherapy (AIT) using recombinant *Culicoides* allergens has been investigated as a preventive measure against IBH in horses. The therapeutic principle of AIT is based on repeated controlled allergen exposure to induce immune deviation from a pathogenic Th2-dominated response toward regulatory and non-inflammatory pathways, accompanied by the induction of allergen-specific IgG antibodies capable of competing with IgE for allergen binding (16, 17). Studies in Icelandic horses in Iceland, and thus naïve to *Culicoides* allergens, have shown that both subcutaneous and intralymphatic administration of recombinant allergens with alum and Monophosphoryl-Lipid A (MPLA) as adjuvants can induce allergen-specific IgG responses and cytokine patterns associated with immunomodulation (18–20). In a therapeutic context, it has been recently demonstrated that AIT with nine major allergens in the same adjuvants (alum/MPLA) led to clinical improvement in IBH-affected horses (21).

Sublingual AIT approaches are well established in human medicine for their safety and propensity to induce tolerance but finds limited use in veterinary science (22, 23).

In our pilot study for a potential sublingual immunotherapy, healthy Icelandic horses were treated with transgenic barley flour expressing the *C. nubeculosus* allergen Cul n 2 using spiral bits. The treatment induced allergen-specific IgG antibodies that were able to partly block IgE-binding and cross-reacted with the corresponding *Culicoides* allergen (Cul o 2) from *C. obsoletus* (24).

The delivery method using spiral bits was not developed further, as the method was not considered to be practical for routine use. Hence, the aim of this study was to develop a more practical method for potential oral immunotherapy using a paste made of barley flour mixed with standard horse feed. To further verify the efficacy of feeding transgenic barley flour as potential oral treatment, we used a major allergen from *C. obsoletus*, Cul o 2p (25), expressed in barley. We evaluated the immunogenicity, IgE-blocking capacity, and cytokine responses following the feeding. We hypothesized that oral administration of this allergen in a simple horse feed mixture would induce allergen-specific immune responses in naïve horses as shown in the pilot study from (24).

## 2 Materials and methods

### 2.1 Animals

Twelve healthy Icelandic horses (age 5–8 years; 4 females, 8 males) in Iceland and thus naïve to IBH-relevant *Culicoides* allergens, were included. Horses were maintained outdoors year-round with hay feeding in winter. Before and after each oral treatment, horses were temporarily housed indoors and restricted from food and water to ensure consistent allergen intake. Veterinary assessments confirmed all animals were clinically healthy prior to inclusion and were regularly monitored throughout the study period. All experiments complied with national animal welfare regulations. The feeding trial in Iceland was conducted under approval from the Icelandic Food and Veterinary Authority (Permit No. 2210671). Cellular antigen stimulation test (CAST) used samples from a separate study in Switzerland, approved by the Animal Experimentation Committee of the Canton of Berne (Permit No. BE 14/20).

### 2.2 Protein production, purification, and functionality assay

#### 2.2.1 Expression and production of recombinant Cul o 2p in barley grains

The expression of the *Culicoides obsoletus* allergen Cul o 2p (NCBI accession number: JX512274) in barley grains was carried out in collaboration with ORF Genetics Ltd. (Kópavogur, Iceland) using the company’s proprietary Orfeus^®^ platform, as previously described (13, 24). The allergen was produced in T3-generation barley grains and detected using Western blotting with Cul o 2p-specific polyclonal antibodies (26). Two stable transgenic lines (line #2 and #25) were selected for mass production. Proteins were purified using the ÄKTA avant chromatography system (Cytiva, Marlborough, MA, USA), followed by tangential flow filtration (TFF) and immobilized metal affinity chromatography (IMAC). Purified *E.coli*-Cul o 2p (11) was used for semiquantitative estimation of Cul o 2p in barley grain as performed according to (24). In short, the concentration of Cul o 2p in barley grain extracts of line #2 and #25 (diluted 1:40) was estimated after Western blot by SynGene – GeneTool for the comparison to a known amount of *E.coli*-Cul o 2p (100 ng and 50 ng) using Cul o 2p specific polyclonal antibody (26) and anti-mouse-HRP (Jackson ImmunoResearch, Ely, United Kingdom).

#### 2.2.2 Cellular antigen stimulation test

The purified barley-produced protein was evaluated using a CAST measuring sulfidoleukotriene release (sLT) from basophils, according to established protocols (26, 27). In total, 45 privately owned horses (age 8–20 years; male n=27, female n=18), healthy (n=19), and IBH-affected (n=26) were tested in CAST with purified Bac-rCul o 2p and barley-rCul o 2p. Bac-rCul o 2p was produced and purified as described before (26).

### 2.3 Preparation of oral allergen mixture

For each feeding, 40 g Cul o 2p barley grains, equivalent to approximately 220 mg rCul o 2p, (ORF Genetics Ltd., Kópavogur, Iceland) were milled into flour and dissolved in 80 mL of a 200 mM NaCl solution (Sigma-Aldrich, St. Louis, MO, USA) (24). Separately, 50 g Fibre-Beet^®^ (British Horse Feeds, Ripon, UK) per horse was soaked in 100 mL tap water (ratio 1:2) to enhance palatability. After incubation for 1 hour at room temperature, mixtures were combined to a paste and administered using standard stable buckets.

### 2.4 Oral treatment and sample collection

**Figure 1:**
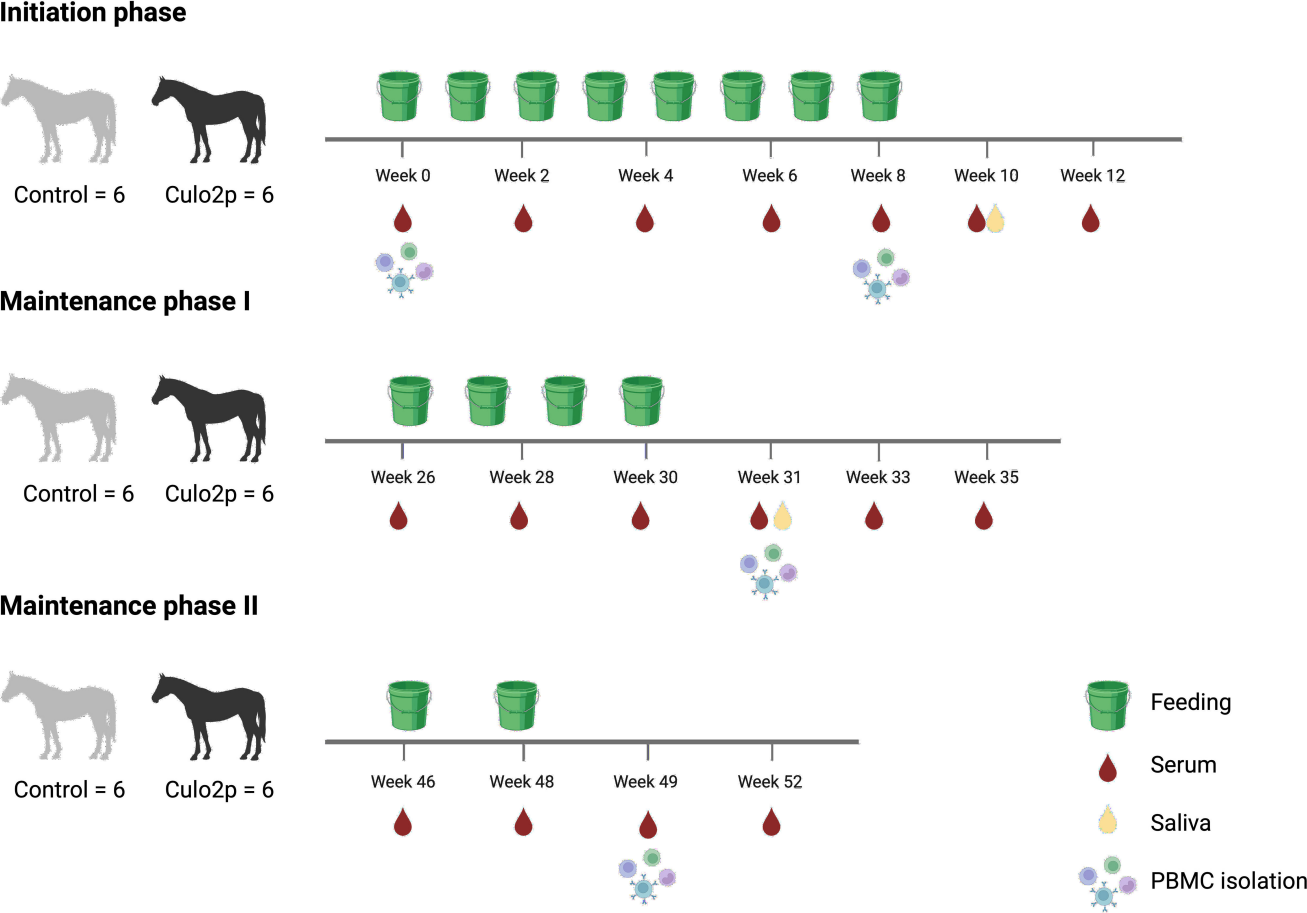
Experimental design of oral immunotherapy with transgenic barley. Twelve healthy Icelandic horses without prior exposure to *Culicoides* allergens were randomly assigned to receive Cul o 2p-expressing barley or control barley (n = 6 per group). The three feeding phases are indicated in green. Horses were bled at multiple time points for collection of serum and isolation of PBMCs. Saliva was taken at weeks 10 and 31. Created with BioRender.com.

The horses were randomly assigned to treatment or control groups (n=6 horses each, based on sex and age). Treatment was carried out in three phases: initiation (weeks 0–8), maintenance phase I (weeks 26–30), and maintenance phase II (weeks 46–48). During the initiation phase, horses were fed five times per week for the first four weeks, followed by three to four times per week over the subsequent four weeks. In maintenance phase I, horses received feedings twice per week for four consecutive weeks. In maintenance phase II, horses were fed five times per week for two consecutive weeks (**Figure 1**).

Six horses received the Cul o 2p transgenic barley mixture made from 40 g barley grain, equivalent to approximately 220 mg of rCul o 2p/horse/feeding, and six horses received standard Golden Promise barley as control group prepared in the same manner. In total, each horse received 52 feedings with 2080 g of barley grain corresponding to 11.44 g rCul o 2p/horse.

Blood serum was collected before and bi-weekly during each phase and up to four weeks post-treatment. Saliva was collected two weeks after the end of the initiation phase (week 10) and maintenance phase I (week 31) and processed according to (24). Peripheral blood mononuclear cells (PBMCs) were isolated from heparinized whole blood collected at weeks 8, 31, and 49 from all study horses as previously described (19, 28).

### 2.5 Cytokine determination in supernatant of *in vitro* restimulated PBMCs

PBMCs were seeded at 0.5 × 10^6^ cells per well in 96-well flat-bottom plates and stimulated with either medium as negative control, recombinant Bac-rCul o 2p (2 µg/mL each) expressed in insect cells using the Bac-to-Bac^®^ Baculovirus expression system (Invitrogen, Carlsbad, CA, USA) (26), or phytohaemagglutinin (PHA, 1 µg/mL; Sigma-Aldrich, Taufkirchen, Germany) as positive control (data not shown). Cells were incubated at 37°C and 5% CO_2_ for 48 hours. Supernatants were collected and stored at –80°C for cytokine analysis.

Cytokine levels (IL-4, IL-10, IFN-γ) were quantified in cell culture supernatants from duplicates using a bead-based multiplex assay, as previously described (29). Spontaneous cytokine secretion was corrected by subtracting the values obtained from the unstimulated (medium-only) cells.

### 2.6 Enzyme-linked immunosorbent assays

#### 2.6.1 Serum ELISA

Allergen-specific IgG, IgG1, IgG4/7, IgG5, IgE, and IgA responses in serum against Cul o 2p were measured by enzyme-linked immunosorbent assay (ELISA) as described before (18). Briefly, ELISA plates were coated with 2 μg/mL rCul o 2p or rCul o 8 protein, an irrelevant control protein, both expressed in *E. coli* (11), blocked with 5% skim milk in PBS-Tween, and incubated with diluted serum samples or controls in duplicate. The use of *E. coli*-expressed antigens ensured that measured antibody responses reflected reactivity to the Cul o 2p protein itself rather than to barley-derived plant components, thereby avoiding potential background reactivity and improving assay specificity.

Serum samples were diluted 1:800 for total IgG (Jackson ImmunoResearch, Ely, United Kingdom), 1:400 for IgG subclasses [IgG1[CVS45], IgG4/7 [CVS 39] (30), and IgG5 [clone 416] (31)], 1:10 for IgA [clone BVS2 (32)], and 1:5 for IgE [clone 3H10 (33)], with optimal dilutions estimated by previous titration assays (data not shown). Negative controls (blocking buffer) and positive controls (sera from horses with high Cul o 2p-specific titers) were included on each plate, alongside reference sera to ensure inter-assay consistency. Antibodies were detected using the corresponding mouse monoclonal antibodies, followed by alkaline phosphatase-conjugated goat-anti mouse IgG polyclonal antibody and developed with phosphatase substrate in 10% diehtanolamine (Sigma-Aldrich, St. Louis, MO, USA). Absorbance was read at 405 nm. Results are shown as ELISA increments, calculated by subtracting baseline optical density (OD) values (week 0) from the OD values obtained.

#### 2.6.2 Saliva ELISA

Saliva samples collected at weeks 10 and 31 were assessed for IgG1, IgG4/7, IgG5, and IgA using the same protocol as for serum, with samples diluted 1:2. Values are presented as ELISA increment compared to baseline.

#### 2.6.3 Blocking ELISA for demonstration of blocking capacity of the induced antibodies

To evaluate the blocking capacity of serum antibodies induced by treatment, a blocking ELISA was performed as previously described (18, 24) with minor modifications. Briefly, ELISA plates were coated with *E. coli*-Cul o 2p (2 µg/mL), blocked, and incubated with pooled serum samples from the two experimental groups at different dilutions (undiluted, 1:2, 1:4, and 1:8), collected at weeks 0, 8, 26, 31, 46, and 49 before serum from an IBH affected horse with Culo2p specific IgE was added. Percent inhibition of IgE binding was calculated using the formula from Jonsdottir et al. (18).

### 2.7 Data analysis

Statistical analyses were performed using GraphPad Prism (GraphPad Software, Boston, MA, USA). Given the small sample size and confirmed non-normal distribution by the Shapiro-Wilk test, non-parametric tests were applied. Differences in antibody levels and cytokine responses between treatment and control groups at selected time points were assessed by Mann-Whitney U tests. To account for multiple comparisons, a Bonferroni correction was applied. Differences were considered statistically significant at *P* ≤ 0.05. Paired comparisons of CAST responses of IBH-affected horses to barley- and baculovirus-expressed proteins were evaluated using the Wilcoxon matched-pairs signed-rank test.

## 3 Results

### 3.1 Production of rCul o 2p in barley grain

**Figure 2:**
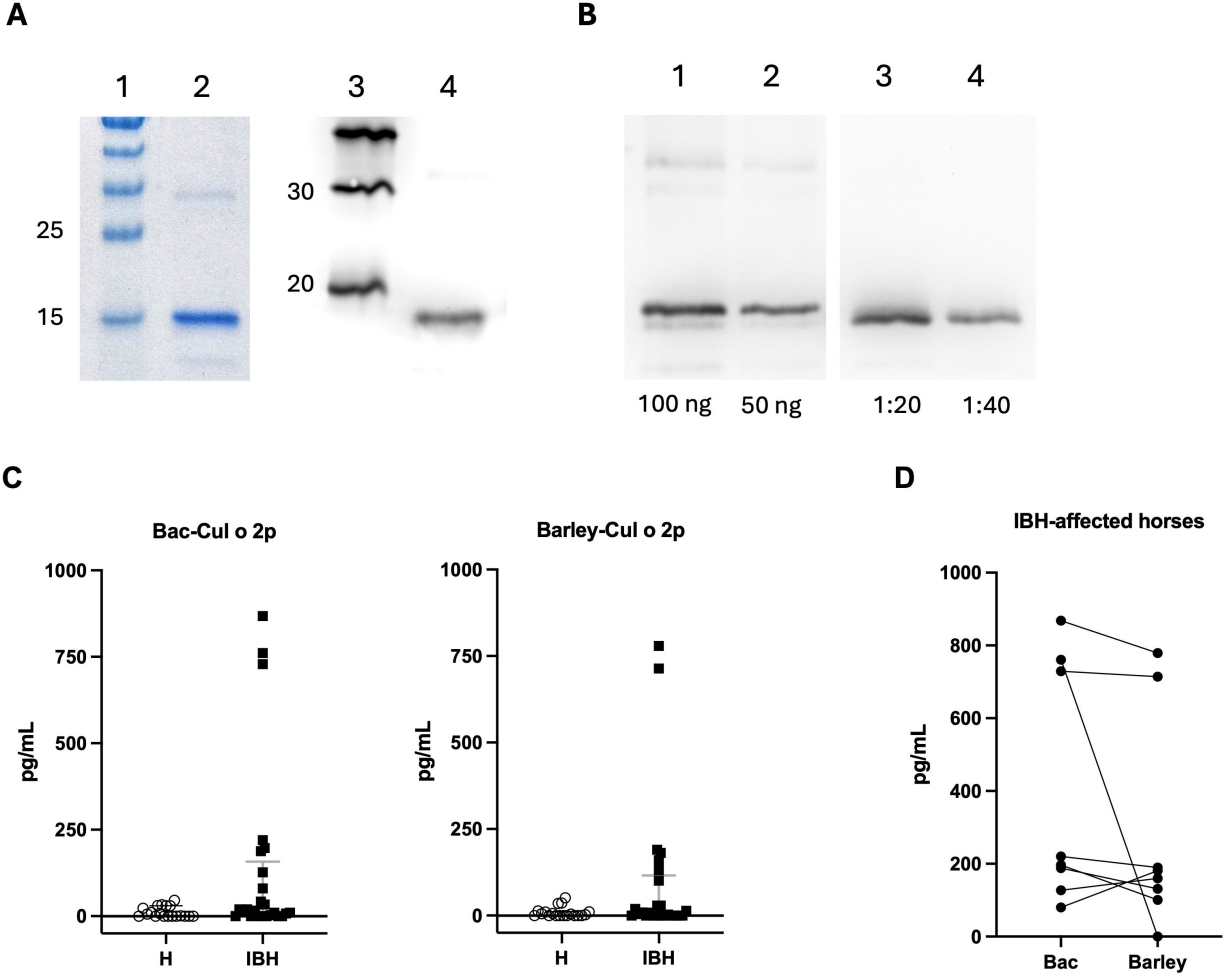
Expression and functional assessment of recombinant Cul o 2p from transgenic barley grains. **(A)** SDS-PAGE stained with Coomassie: PageRuler™ Prestained Protein Ladder (lane 1), barley-expressed rCul o 2p extract (50 ng; lane 2). Western blot using Cul o 2p-specific polyclonal antibody: MagicMark™ XP Western Protein Standard (lane 3), purified rCul o 2p from transgenic barley line #25 (lane 4). **(B)** Western blot after protein extraction from milled barley grains with 200 mmol NaCl: *E*. *coli*-expressed rCul o 2p (100 ng; lane 1), *E*. *coli*-expressed rCul o 2p (50 ng; lane 2), barley-expressed rCul o 2p from line #25 diluted 1:20 (lane 3) and 1:40 (lane 4). **(C)** Sulfidoleukotriene (sLT) release from peripheral blood leukocytes of healthy (H = 19) and IBH-affected horses (IBH = 26) after *in vitro* stimulation with Bac- and barley-expressed rCul o 2p. Mann–Whitney U test was used for statistical analysis between the groups. No statistical differences were detected. **(D)** Paired comparison of sLT release in IBH-affected, Cul o 2p-positive horses after stimulation with Bac- versus barley-derived rCul o 2p. Wilcoxon test was used for statistical analysis between the groups. No statistical differences were detected. Created with BioRender.com.

Expression of recombinant Cul o 2p in transgenic barley grain was confirmed by SDS-PAGE and Western blot analysis. Coomassie staining revealed a distinct band at the expected molecular weight (~17 kDa) in extracts from barley line #25. Western blotting with an anti-His antibody further confirmed the presence of His-tagged rCul o 2p in extracts from barley line #25 (**Figure 2A**). To estimate the concentration of Cul o 2p in barley grain, a dilution series of barley-rCul o 2p line #25 was compared to *E. coli*-expressed rCul o 2p, yielding an approximate quantification of 5.1 mg Cul o 2p per gram of grain (**Figure 2B**). Similar expression levels were observed in barley line #2 with 5.9 mg Cul o 2p per gram of grain (data not shown), resulting in an overall average of 5.5 mg/g across both lines.

The allergenic activity of purified barley-rCul o 2p was assessed using CAST with peripheral blood leukocytes from IBH-affected and healthy horses. Stimulation with both Bac- and barley-derived rCul o 2p induced sLT release in IBH-affected horses, while responses in healthy controls remained low (**Figure 2C**). Among IBH horses, 8/26 responded to Bac-rCul o 2p and 7/26 to barley-rCul o 2p, with seven individuals responding to both. Comparison of responses within IBH horses showed no significant difference in sLT release between Bac- and barley-derived rCul o 2p (**Figure 2D**).

### 3.2 Cul o 2p-specific responses in sera and saliva following oral treatment with transgenic barley

#### 3.2.1 Serum

Allergen-specific responses were measured in serum over the course of the study. After treatment with rCul o 2p-transgenic barley, four out of six horses in the treatment group had induced allergen-specific levels of IgA and IgG, mainly IgG1 and IgG4/7. These horses were effectively boosted during both maintenance phase I and II.

**Figure 3:**
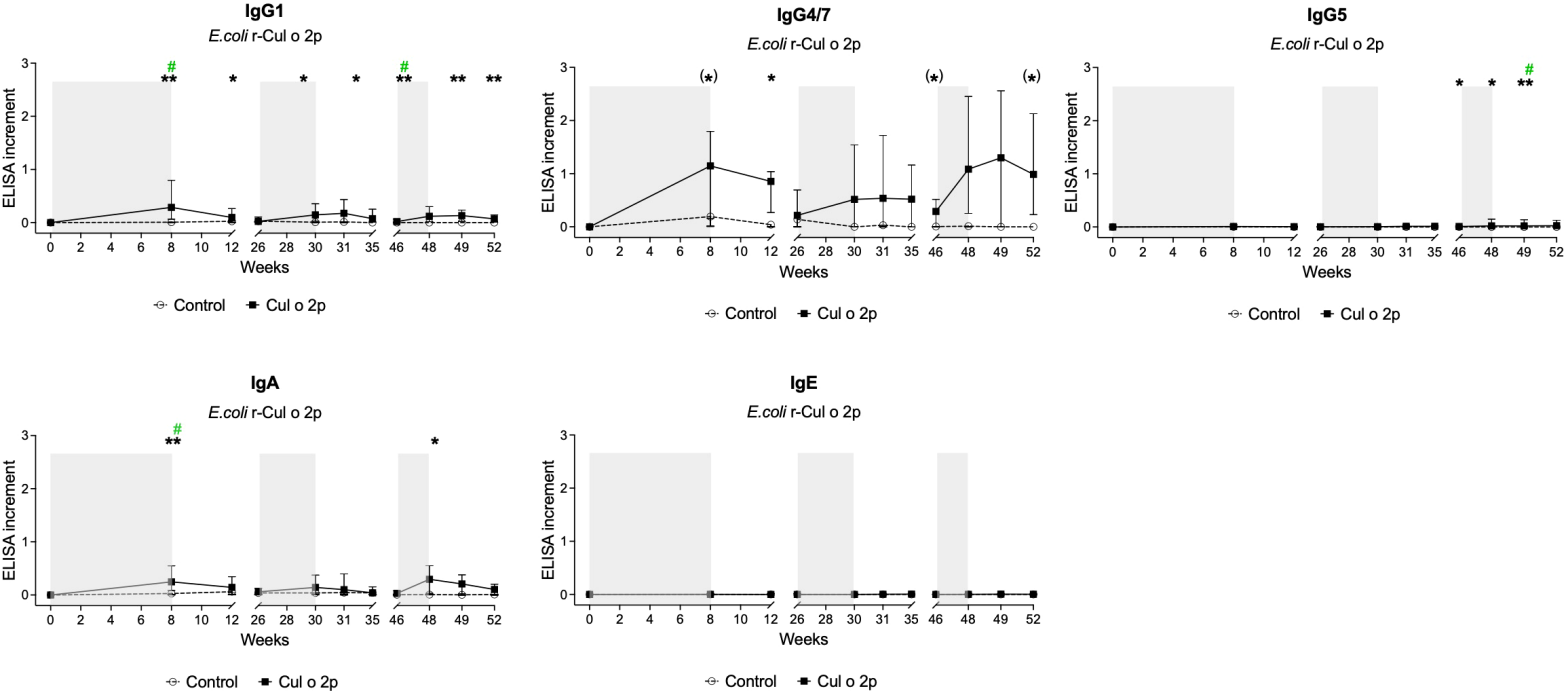
Allergen-specific antibody responses in serum following oral treatment with Cul o 2p barley. Cul o 2p specific serum levels of IgG subclasses (IgG1, IgG4/7, IgG5), IgA, and IgE measured by ELISA. Grey areas indicate the feeding phases: initiation phase, maintenance phase I, and maintenance phase II. Results are shown as ELISA increment with median and interquartile range for the control (○; n=6) and Cul o 2p- horses (■; n=6). Mann–Whitney U test was used for statistical analysis between groups at each time point. p-values were corrected for multiple comparisons using the Bonferroni correction. Asterisks indicate statistical significance *p< 0.05; **p< 0.01; ***p< 0.001*;* # (green) after Bonferroni correction. Trends toward significance (p< 0.1) are reported as (*).

Mann-Whitney U test comparisons between treatment and control groups showed significant differences across the measured immunoglobulin isotypes at distinct time points. After applying Bonferroni correction for multiple testing (α = 0.0045), significant differences remained for IgG1 at week 8 (end of the initiation phase) and week 46 (prior to maintenance phase II), for IgG4/7 at week 49 (after maintenance phase II), and for IgA at week 8 (**Figure 3**).

IgG antibodies in serum were first detected four weeks into the initiation phase (data not shown). Induced IgG4/7 levels stayed elevated post treatment compared to IgG1 and IgA levels which declined immediately after cessation of feeding. A similar response pattern was observed during the maintenance phases. IgG5 responses were not detectable after initiation or maintenance phase I. A minor increase in IgG5 levels was observed during maintenance phase II, primarily driven by a single individual. No IgE reactivity was detected in any horse throughout the study.

**Figure 4:**
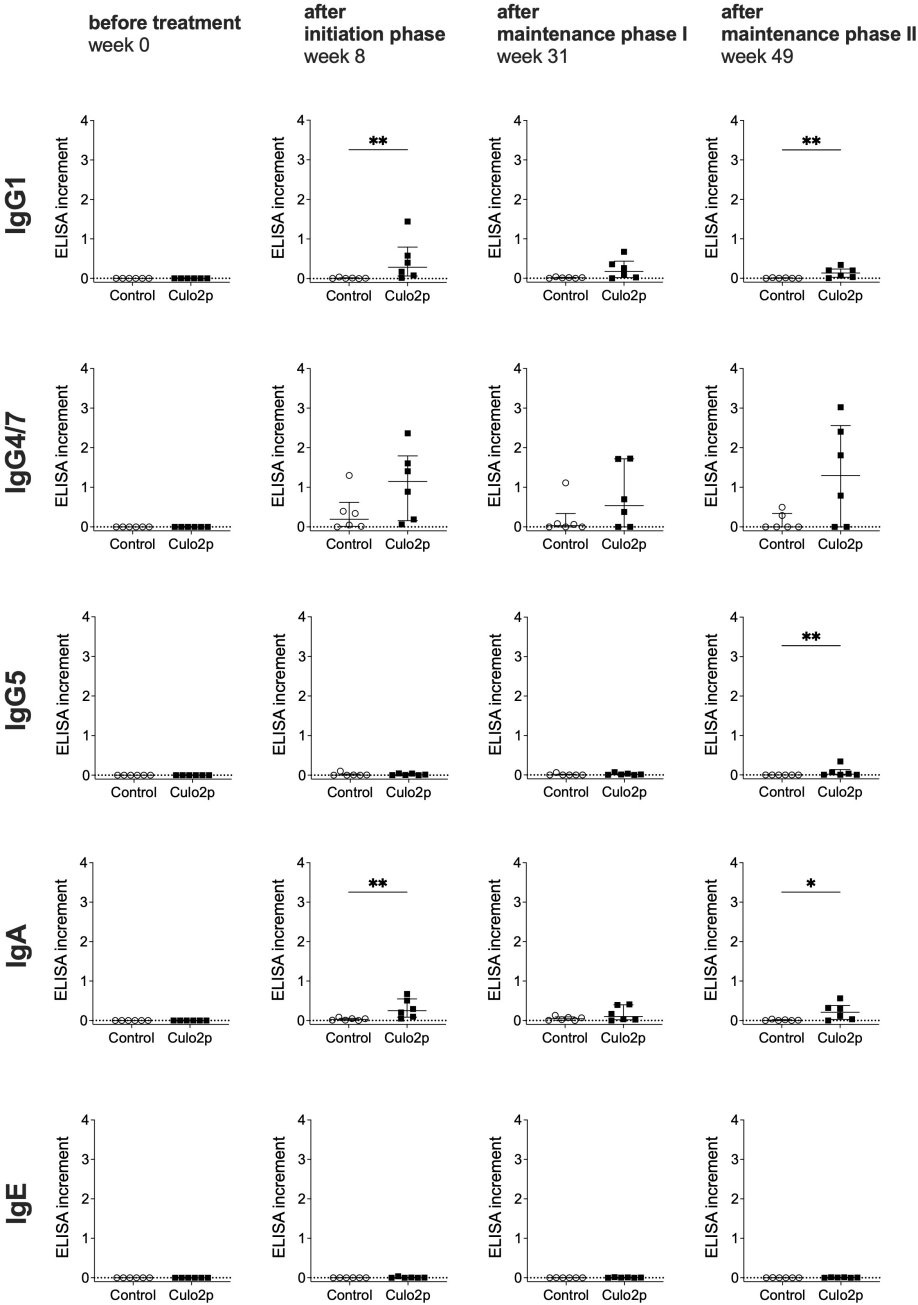
Individual allergen-specific antibody responses in serum following oral treatment with Cul o 2p barley. Individual Cul o 2p specific serum levels of IgG subclasses (IgG1, IgG4/7, IgG5), IgA, and IgE measured by ELISA. Results are shown as ELISA increment with median and interquartile range for the control (○) and Cul o 2p- horses (■) before treatment (week 0), after initiation phase (week 8), after maintenance phase I (week 31), and after maintenance phase II (week 49). Mann–Whitney U test was used for statistical analysis between groups at each time point. Asterisks indicate statistical significance *p< 0.05; **p< 0.01; ***p< 0.001.

Individual Cul o 2p–specific antibody responses were compared between the two groups at selected time points (weeks 0, 8, 31, and 49), corresponding to the end of the initiation phase and both maintenance phases, using the Mann–Whitney U test. Significant difference was observed between the groups at the end of the initiation phase (week 8) for IgG1 and IgA, and after maintenance phase II (week 49) for IgG1, IgG5, and IgA. In the control group, low-level Cul o 2p–specific IgG4/7 was observed in a subset of horses. At week 8, 3/6 control horses showed slightly increased IgG4/7 levels, with one individual displaying markedly higher levels than the others. At week 31, IgG4/7 responses were dominated by this single individual (with high levels), whereas the remaining control horses remained low. At week 49, two out of six control horses showed increased IgG4/7 levels (**Figure 4**).

**Figure 5:**
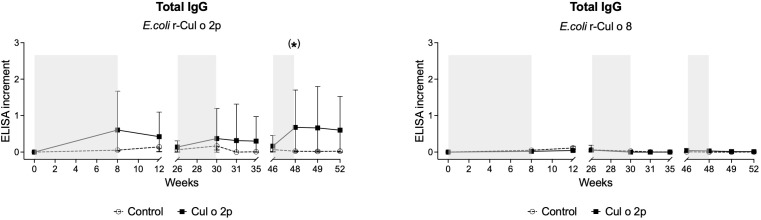
Allergen specificity of IgG responses following oral Cul o 2p treatment. Total IgG serum levels against Cul o 2p and Cul o 8 measured by ELISA. Grey areas indicate the feeding phases: initiation phase, maintenance phase I, and maintenance phase II. Results are shown as ELISA increment with median and interquartile range for the control (○) and Cul o 2p- horses (■). Mann–Whitney U test was used for statistical analysis between groups at each time point. p-values were corrected for multiple comparisons using the Bonferroni correction. Asterisks indicate statistical significance *p< 0.05; **p< 0.01; ***p< 0.001*;* # (green) after Bonferroni correction. Trends toward significance (p< 0.1) are reported as (*).

To confirm specificity of the induced Cul o 2p antibody response, total Cul o 2p IgG levels in serum were measured with *E. coli*-expressed rCul o 2p and the unrelated *Culicoides* allergen rCul o 8 by ELISA. Total anti-Cul o 2p IgG levels were consistent with the observed trend in IgG1 and IgG4/7. No IgG response was detected against Cul o 8 (**Figure 5**).

#### 3.2.2 Saliva

**Figure 6:**
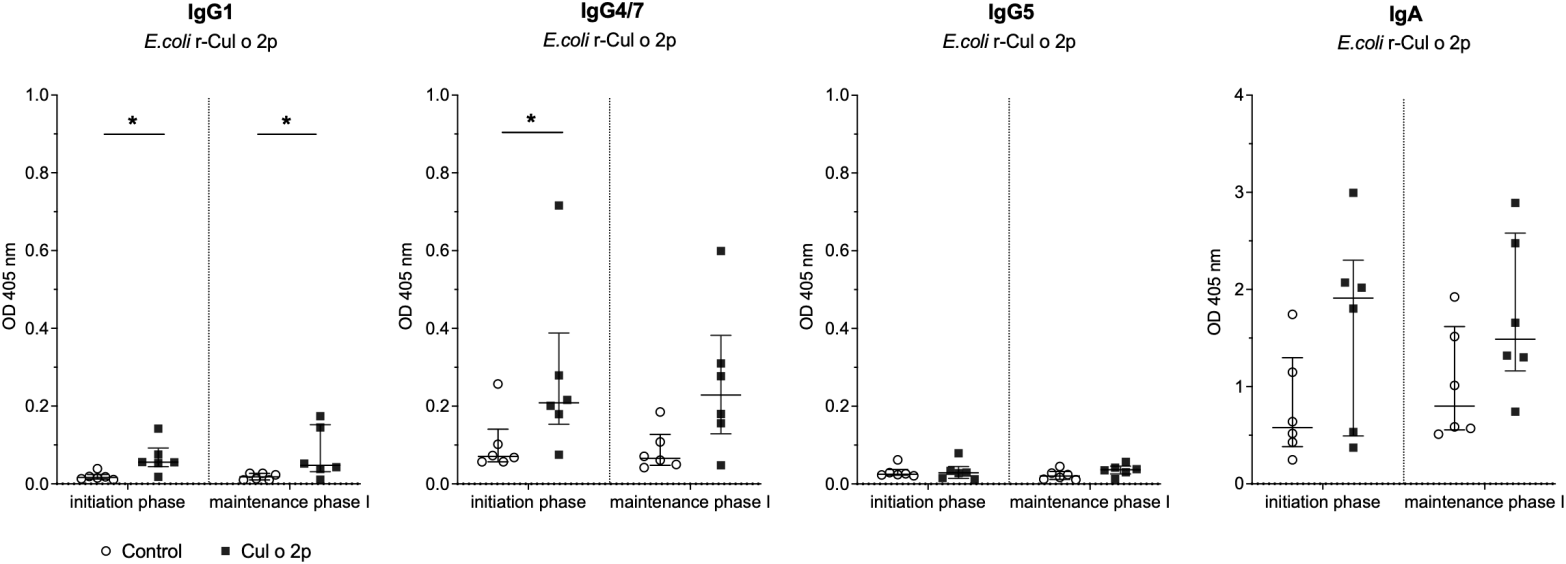
Allergen-specific antibody responses in saliva following oral treatment with Cul o 2p barley. Cul o 2p–specific IgG1, IgG4/7, IgG5, and IgA levels in saliva were measured by ELISA after initiation phase and maintenance phase I. Results are presented for individual horses in each group, control (○), and Cul o 2p-treated (■) as optical density at 405 nm, with median and interquartile range indicated for each group. Mann–Whitney U test was used for statistical analysis between groups at each time point. Asterisks indicate statistical significance *p< 0.05; **p< 0.01; ***p< 0.001.

In addition to systemic antibody responses, mucosal responses were assessed by measuring Cul o 2p-specific IgG1, IgG4/7, IgG5, and IgA levels in saliva after the initiation phase and maintenance phase I. Five out of six treated horses developed measurable salivary IgG1 and IgG4/7 responses following the initiation phase and the same individuals were efficiently boosted after maintenance phase I. No IgG5 response was detected at either time point. IgA responses were observed in four of six horses post-initiation and in five of six horses following maintenance phase I. However, non-specific background reactivity was also detected in control animals, particularly for IgA. Statistically significant differences between the treatment and control groups were observed for IgG1 and IgG4/7 after the initiation phase, and for IgG1 after maintenance phase I (*p* < 0.05; Mann-Whitney U test; **Figure 6**).

### 3.3 Induced Cul o 2p-specific antibodies inhibit IgE binding in sera following oral treatment with transgenic barley

**Figure 7:**
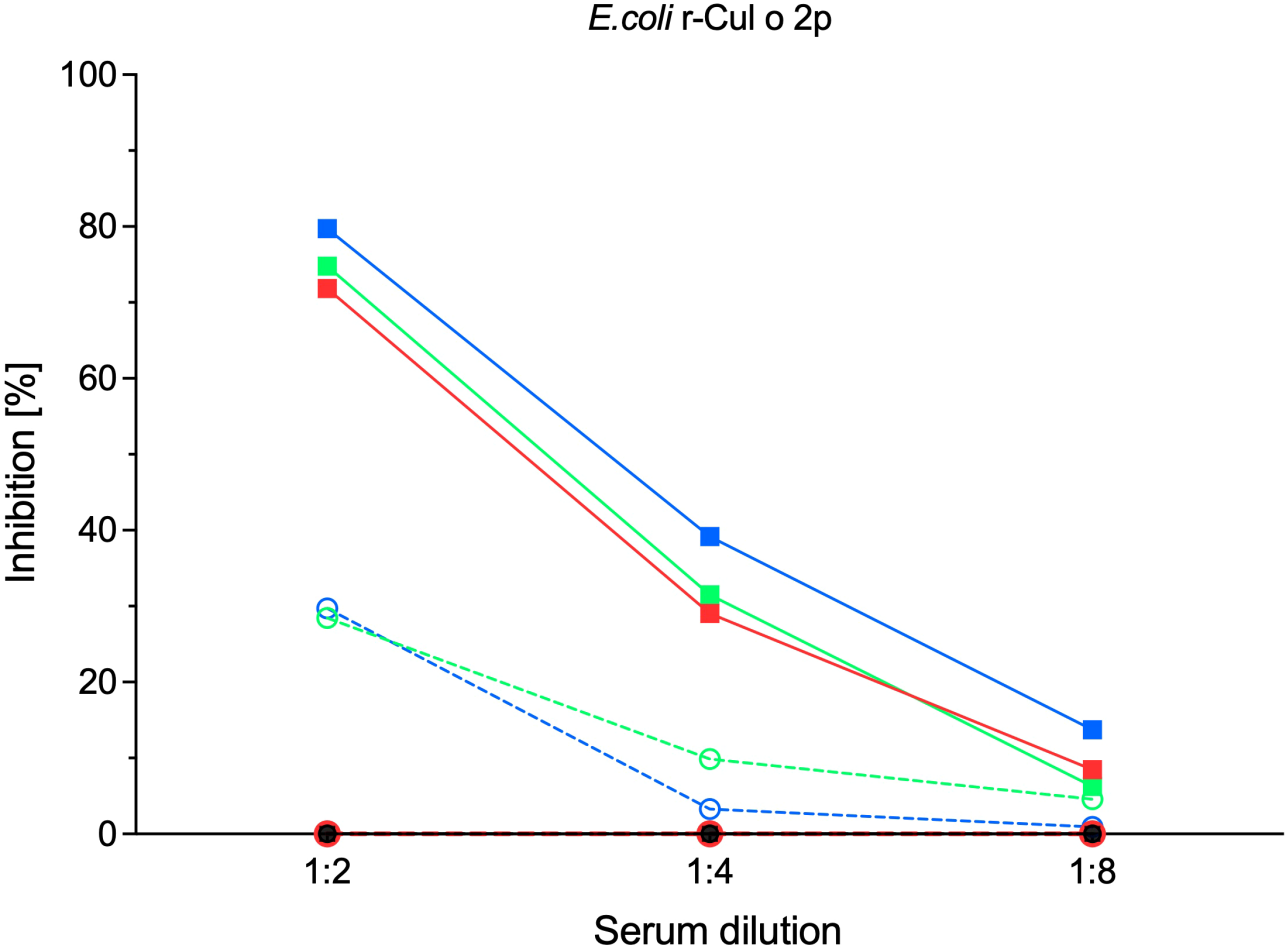
Induced Cul o 2p-specific antibodies inhibit IgE binding in sera following oral treatment. Pooled serum from control (○-–) and Cul o 2p treated (■**^:_^**) horses diluted 1:2, 1:4 and 1:8, at four different time points were applied to an ELISA plate coated with *E.coli-*rCul o 2p prior to addition of serum from an IBH positive horse at dilution 1:5. The inhibition (%) is shown as mean for the control (○), and Cul o 2p-treated (■) horses before treatment (black), after initiation phase (red), after maintenance phase I (green), and after maintenance phase II (blue).

The blocking capacity of serum antibodies induced by the treatment was assessed using a blocking ELISA, in which pooled sera were pre-incubated with *E. coli*-expressed rCul o 2p prior to the addition of serum from an IBH-affected horse. Sera from Cul o 2p-treated horses effectively inhibited IgE binding, whereas sera from control horses fed normal barley showed only minimal inhibition. Inhibitory effects were detectable after the initiation phase and increased with each subsequent treatment. Mean inhibition levels after the initiation phase were 72%, 29%, and 9% at serum dilutions of 1:2, 1:4, and 1:8, respectively. A comparable but slightly higher inhibitory effect was observed after the first maintenance phase. The highest blocking capacity was detected following maintenance phase II, with 80%, 39%, and 14% inhibition at the respective dilutions. In contrast, control sera showed minimal IgE inhibition at all time points, with only 30%, 3%, and 1% inhibition observed after maintenance phase II (**Figure 7**). Statistical analysis was not performed due to the use of pooled samples.

### 3.4 Cytokine detection following *in vitro* re-stimulation of PBMCs with insect cell produced Bac-rCul o 2p

Cytokine secretion was assessed by *in vitro* re-stimulation of PBMCs with Bac-rCul o 2p at weeks 8, 31, and 49, corresponding to the end of each phase. IL-10, IL-4, and IFN-γ were measured in the cell culture supernatants.

Following the initiation phase, IFN-γ levels were slightly elevated with high inter-individual variability in the Cul o 2p-treated group (median=3.9 [0–42] U/mL) compared to controls (median=3.1 [1.8–4.9] U/mL), while IL-10 and IL-4 levels remained low (IL-10: control=1.3 [0.3–2.7] pg/mL, treated=0.4 [0–4.5] pg/mL; IL-4: control=3.5 [1.7–4.5] pg/mL, treated=0.8 [0–3.1] pg/mL). After maintenance phase I, a significant increase in IFN-γ was observed in the treatment group (median=63.4 [42.5-453.5] U/mL) relative to controls (median=3.9 [0-51.3] U/mL), although one control horse showed elevated IFN-γ secretion. IL-10 levels increased in both groups, with slightly higher median concentrations in treated horses (treated=38 [22.5–58] pg/mL, control=14.6 [4.3–33.7] pg/mL) but the difference did not reach significance. IL-4 remained low across both groups.

**Figure 8:**
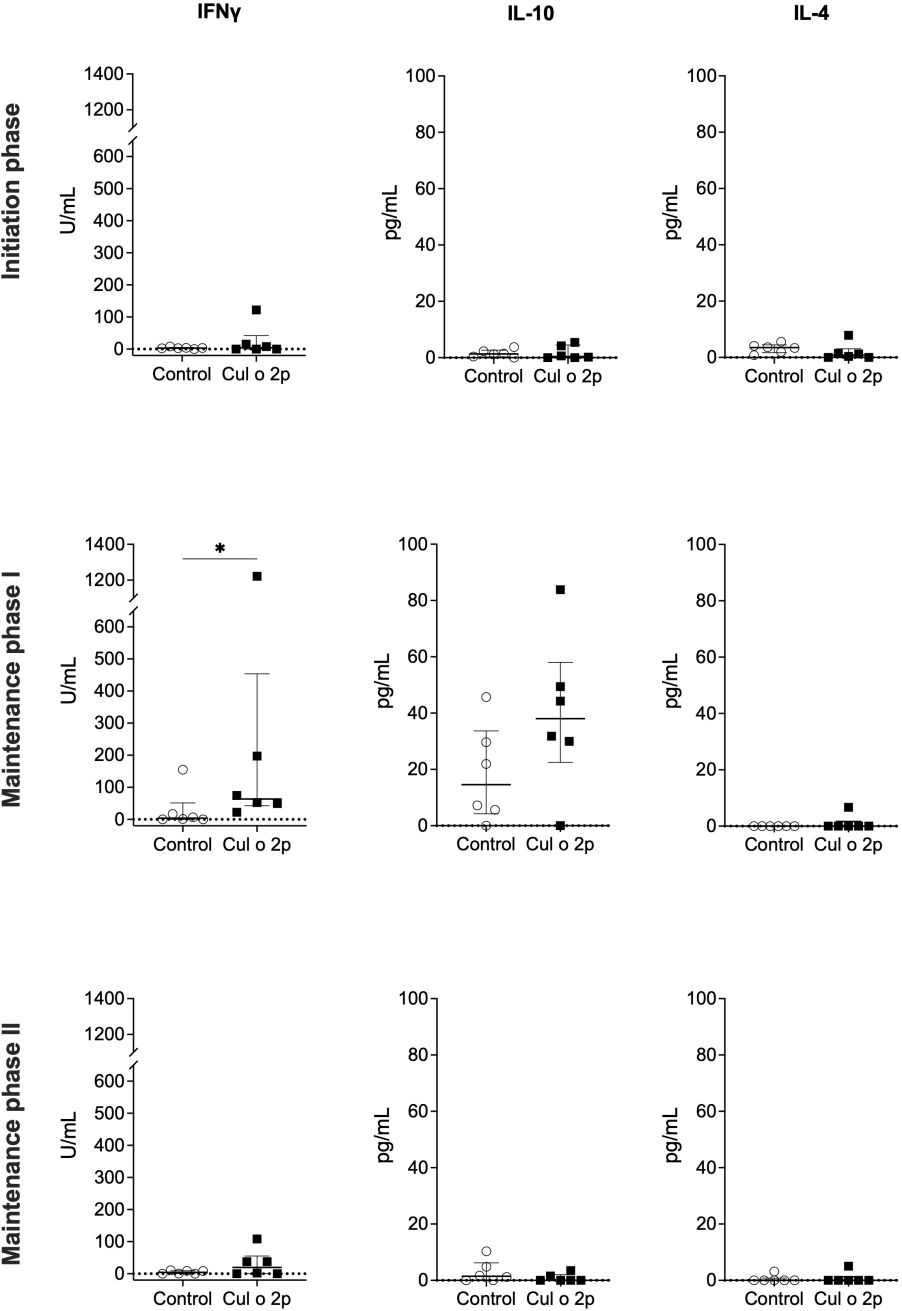
Cytokine secretion after *in vitro* restimulation of PBMCs with Bac-Culo2p. PBMCs from control (○) and Cul o 2p-treated horses (■) were isolated and stimulated *in vitro* with Bac-rCul o 2p. IFNγ, IL-10, and IL-4 were measured in cell culture supernatants using a bead-based multiplex immunoassay. Cytokine responses are shown for individual horses in both groups following the initiation phase, maintenance phase I, and maintenance phase II. Data are presented as median with interquartile range for each group. Correction for spontaneous cytokine release was made by subtracting values from the medium. Mann–Whitney U test was used for statistical analysis between groups at each time point. Asterisks indicate statistical significance *p< 0.05.

At the end of maintenance phase II, IFN-γ levels in the treatment group remained slightly elevated compared to the control group. IL-10 and IL-4 levels were low in both groups, with slightly higher IL-10 concentrations observed in control horses (**Figure 8**).

## 4 Discussion

This study demonstrates that oral administration of barley expressing Cul o 2p in a feed-based formulation can induce allergen-specific immune responses in naïve Icelandic horses. Four of six treated horses developed Cul o 2p-specific IgG1, IgG4/7, and IgA in serum and saliva, with IgG4/7 persisting beyond the feeding period. Pooled sera exhibited >70% IgE-blocking capacity. Upon allergen re-stimulation, IFN-γ secretion was significantly increased in treated compared to control horses. IL-10 secretion was also increased but the difference between the groups did not reach statistical significance (p=0.14 after maintenance phase I), so definitive conclusions cannot be drawn. Nevertheless, the data suggest a Th1-biased and regulatory immune response to the barley allergen. Importantly, there was no detectable Cul o 2p-specific IgE or IgG5, nor elevated IL-4, suggesting this approach did not promote allergic sensitization.

A previous pilot study by Jonsdottir et al. (24) explored a sublingual-like route for induction of allergen-specific antibodies by using transgenic barley to deliver a *Culicoides* allergen. Horses were treated with transgenic barley using spiral bits to prolong contact with the oral mucosa to mimic the prolonged sublingual retention of allergen in human sublingual immunotherapy (SLIT), where patients typically hold the tablet under the tongue for a few minutes before swallowing (34, 35). While the study successfully induced specific IgG and IgA responses, the reliance on specialized equipment and prolonged feeding times underscored the impracticality of this method in routine stable management.

The present study represents a pragmatic shift toward a model more analogous to oral immunotherapy (OIT). These two modalities engage the mucosal immune system differently. SLIT relies on extended contact with the sublingual mucosa, where a favorable safety profile is achieved through localized allergen uptake that minimizes systemic exposure (34, 36). In contrast, OIT involves swallowing the allergen, leading to brief oral engagement followed by immunological processing primarily in the gut-associated lymphoid tissue (GALT) (35, 37). To achieve robust desensitization, OIT requires significantly larger allergen doses, which results in stronger clinical and immunological changes than SLIT, but at the cost of a higher incidence of side effects (38–40).

This study introduces a feed-based approach as a simple, scalable alternative to invasive subcutaneous injections, aligning with the trend toward non-invasive oral immunotherapies (like SLIT and OIT), which are favored for their enhanced safety and ease of use (41).

We analyzed systemic (serum) and mucosal (saliva) antibody responses together with cytokine secretion to evaluate the safety and immunogenicity of the feed-based approach in naïve horses. While various allergens could theoretically elicit immune responses, the use of a major allergen maximizes clinical and translational relevance for future applications in IBH. The induction of Cul o 2p-specific IgG1, IgG4/7, and IgA, along with increased IL-10 and IFN-γ secretion, demonstrates that this approach is indeed immunogenic, without evidence of *de novo* IgE sensitization.

In human AIT, the induction of allergen-specific IgG isotypes is a key marker of tolerance, modifying the immunological response to the allergen (16). Allergen-specific IgG1 typically rises first and is critical for early IgE-blocking activity, often serving as the dominant inhibitory isotype in the initial phase of treatment (42). The subsequent development of IgG4 is considered the hallmark of long-term tolerance, requiring sustained IL-10 signaling from regulatory T cells to drive class switching and establish the dominant, long-lasting “blocking antibody” response (16, 42, 43). Functionally, these IgG antibodies compete with IgE for allergen binding, thereby preventing cross-linking of FcϵRI on mast cells and basophils and suppressing effector cell activation (16, 17, 43, 44). However, equine IgG subclasses are not direct functional analogues of human IgG1 and IgG4, and similar IgG patterns in horses cannot be assumed to mediate identical mechanisms of tolerance (45–47). Subclass profiles may nevertheless offer context-dependent clues about underlying immune pathways, as described for Th1- and Th2–associated responses in horses (48).

In our study, we observe an early rise of allergen-specific IgG1 levels with a rapid decline after feeding ceased, while IgG4/7 persisted beyond the feeding period. IgG1 in horses is frequently induced early after antigen exposure, including during viral infection, tetanus vaccination, and in allergic responses alongside IgE, but it is not consistently associated with a specific T-helper profile and its interpretation is therefore context-dependent (46, 48–50). In contrast, IgG4/7 is considered a Th1–associated isotype and is prominently induced during viral infections such as EHV-1 and equine influenza, within which it parallels IFN-γ–dominated Th1 responses and has been associated with protective immunity (47, 48, 51–53). In line with this immunological context, treated horses in our study showed elevated IL-10 and IFN-γ upon allergen re-stimulation, suggesting engagement of regulatory and Th1-associated pathways. The slower decline of Cul o 2p–specific IgG4/7 compared to IgG1 is therefore compatible with a systemic antibody response that extends beyond the feeding period, although the durability or protective relevance of this response was not assessed.

Low-level Cul o 2p–specific IgG4/7 reactivity was detected in a subset of control horses, with one individual displaying markedly higher titers at certain time points. As no horse-biting *Culicoides* species are present in Iceland, these responses are unlikely to reflect natural allergen exposure to *Culicoides*. It might be due to cross-reactivity between *Simulium* and *Culicoides* saliva proteins, as *Simulium* occur in Iceland and bite horses or they may be attributable to antibodies directed against *E. coli*–derived components present in the recombinant Cul o 2p used for serology.

Importantly, the absence of Cul o 2p-specific IgE, together with low or no IgG5–an isotype associated with allergic sensitization in horses (54–56)–and the lack of IL-4 upregulation do not suggest that this approach promoted a Th2-mediated or allergic response. Moreover, the induced IgG antibodies partially blocked IgE binding to sera from IBH-affected horses, providing functional evidence of blocking capacity.

The induction of allergen-specific IgA further supports immunogenicity of the approach, as IgA is a recognized marker of AIT in humans (17, 57) and in horses it represents a key mucosal isotype involved in barrier immunity and early antigen neutralization (32, 46, 58).

We observed two non-responder horses that failed to induce Cul o 2p-specific antibodies in serum and saliva, lacked IgE-blocking capacity (individual data not shown), and showed weak IL-10 and IFN-γ responses. Such inter-individual variability in immunological responsiveness is well recognized and widely reported in AIT studies, with significant differences documented not only in the magnitude of the overall response but also in the specific cytokine and antibody profiles generated between individuals, even within a single experimental group (43, 59, 60).

The detection of IgG1, IgG4/7, and IgA in saliva indicates effective engagement of the oral mucosa, while their presence in serum indicates a systemic activation. Together, these findings demonstrate that the feed-based approach induces a coordinated mucosal and systemic immune response. Comparable antibody profiles were observed in the initial study by Jonsdottir et al. (24), where oral administration via spiral bits induced IgG1 and IgG4/7 responses in all treated horses (n = 4). Although the two studies used different allergens and inhibition assays with varying dilutions, both demonstrated IgE-blocking activity, suggesting comparable immunological outcomes despite methodological differences.

A key strength of this study is the development of a simple, feed-based approach. By using barley expressing Cul o 2p, the allergen was incorporated directly into feed without purification, offering a straightforward, scalable, and cost-effective model for production, storage, and administration. This approach overcomes challenges from previous oral delivery attempts in horses that required prolonged mucosal contact with specialized equipment (24). Here, we adapted this method by feeding larger quantities of transgenic barley, resulting in higher allergen intake compared to the spiral-bit approach. However, direct comparison between the two studies is not applicable, as different allergens were used.

The small sample size (n=6) limits statistical power and generalizability, and the use of immunologically naïve Icelandic horses does not reflect the complex sensitization patterns of IBH-affected animals.

A major allergen was tested in this pilot study. Although IBH is characterized by co-sensitization to multiple allergens (7), the immediate priority is to establish whether a feed-based oral immunotherapy protocol is safe and effective in IBH-affected horses. Once feasibility and clinical benefit have been demonstrated in allergic animals, future approaches may build on this platform by incorporating additional allergens to more comprehensively reflect the immunopathogenesis of IBH and enhance clinical efficacy. The critical next step will therefore be a proof-of-concept placebo-controlled clinical trial in IBH-affected horses to assess efficacy. Beyond IBH, barley expressing allergens as an oral delivery platform represents a promising tool for developing practical and scalable oral immunotherapies across veterinary and human medicine.

## 5 Conclusion

We conclude that the feed-based approach with barley expressing Cul o 2p represents a platform that can induce regulatory and Th1-associated immune responses in naïve horses without promoting allergic sensitization. If clinical efficacy can be demonstrated, this OIT strategy offers a practical, scalable, and non-invasive option for IBH management.

## Data Availability

The original contributions presented in the study are included in the article/supplementary material. Further inquiries can be directed to the corresponding author/s.
